# A cause to consider for chronic unresolving diarrhea

**DOI:** 10.1093/gastro/gou071

**Published:** 2014-10-16

**Authors:** Talal Hilal

**Affiliations:** Department of Internal Medicine, University of Kentucky, Lexington, Kentucky, USA

**Keywords:** chronic diarrhea, carcinoid syndrome, neuroendocrine tumor, hepatomegaly

## Abstract

A 36-year-old male who carried a diagnosis of irritable bowel syndrome presented with chronic watery diarrhea and was found to have hepatomegaly on physical exam. A computed tomography (CT) scan of the abdomen revealed hepatomegaly with lesions suspicious for metastatic disease. A colonoscopy revealed a polypoid lesion in the terminal ileum, which was biopsied, revealing a neuroendocrine tumor (NET). He was treated with palliative octreotide and chemoembolization of liver metastases until disease progression. The case highlights the importance of considering functional NETs, especially carcinoid syndrome, in patients with chronic unresolving diarrhea, since early diagnosis allows for further treatment options that can prolong survival.

## Introduction

There are numerous causes of chronic diarrhea, some of which are uncommon. Carcinoid syndrome is one of those uncommon causes that develop when a gastrointestinal (GI) neuroendocrine tumor (NET) metastasizes to the liver and releases serotonin and other mediators into the systemic circulation. This occurs in less than 10% of all NET cases, and the main clinical features are diarrhea, cutaneous flushing, and dyspnea [1]. Our case seeks to highlight the importance of considering functional NETs, especially carcinoid syndrome, in patients presenting with chronic unresolving diarrhea.

## Case presentation

A 36-year-old male presented to his primary care physician with episodic watery diarrhea and urgency that had been gradually worsening over a 2-year period. He denied abdominal pain, bloody bowel movements, skin rashes or ulcerations, but reported a recent 10 kg weight loss. He was diagnosed with irritable bowel syndrome when these symptoms started 2 years prior to the current presentation. At the time, he had a negative work-up, including a normal colonoscopy, and was given loperamide for symptomatic management. Past medical, surgical, social and family histories were unremarkable. He was not on any medications apart from loperamide and was not known to have any allergies.

Physical examination revealed vital signs that were within normal limits, apart from tachycardia and tachypnea, with a temperature of 36.7°C, heart rate of 122 beats per min, blood pressure of 109/79 mmHg, respiratory rate of 22/min with oxygen saturation of 98% on room air. The patient was a well-appearing male in mild distress. His skin and mucous membranes were dry, without rashes. Lung auscultation revealed normal vesicular breath sounds bilaterally without wheezes or crepitations. Heart auscultation revealed normal S1 and S2 without murmurs. Abdominal exam revealed massive hepatomegaly with a palpable, non-tender liver edge in the right iliac fossa.

**Figure 1.**
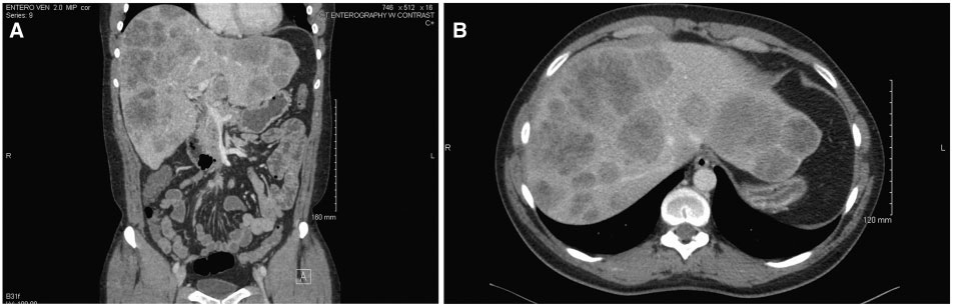
(A) Coronal and (B) axial slices through the abdomen, showing hepatomegaly with multiple lesions within the liver parenchyma.

**Figure 2.**
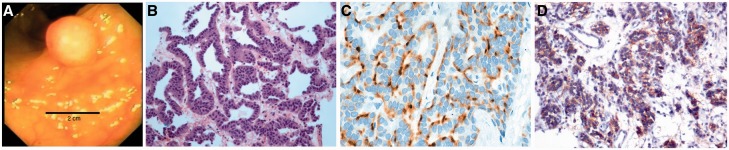
(A) Polypoid lesion in the terminal ileum seen on colonoscopy. (B) Hematoxylin and eosin stain of biopsy specimen, showing a well-differentiated neuroendocrine tumor. (C) Immunohistochemistry for chromogranin A and (D) for synaptophysin, confirming diagnosis of a neuroendocrine tumor.

Laboratory data were unremarkable, including hemoglobin, liver enzymes, stool analysis and culture, stool ova and parasites and polymerase chain reaction (PCR) for *Clostridium difficile* in stool sample. Abdominal computed tomography (CT) with intravenous contrast revealed massive hepatomegaly with multiple liver hyperintensities (Figures 1A and 1B), a normal-appearing colon, and no identifiable lymphadenopathy. Due to the metastatic appearance of the liver lesions, the patient was scheduled for a colonoscopy. The procedure did not reveal gross abnormalities in the colon, but terminal ileoscopy showed a small, 2 cm, polypoid lesion in the terminal ileum, which was biopsied (Figure 2A).

**Figure 3.**
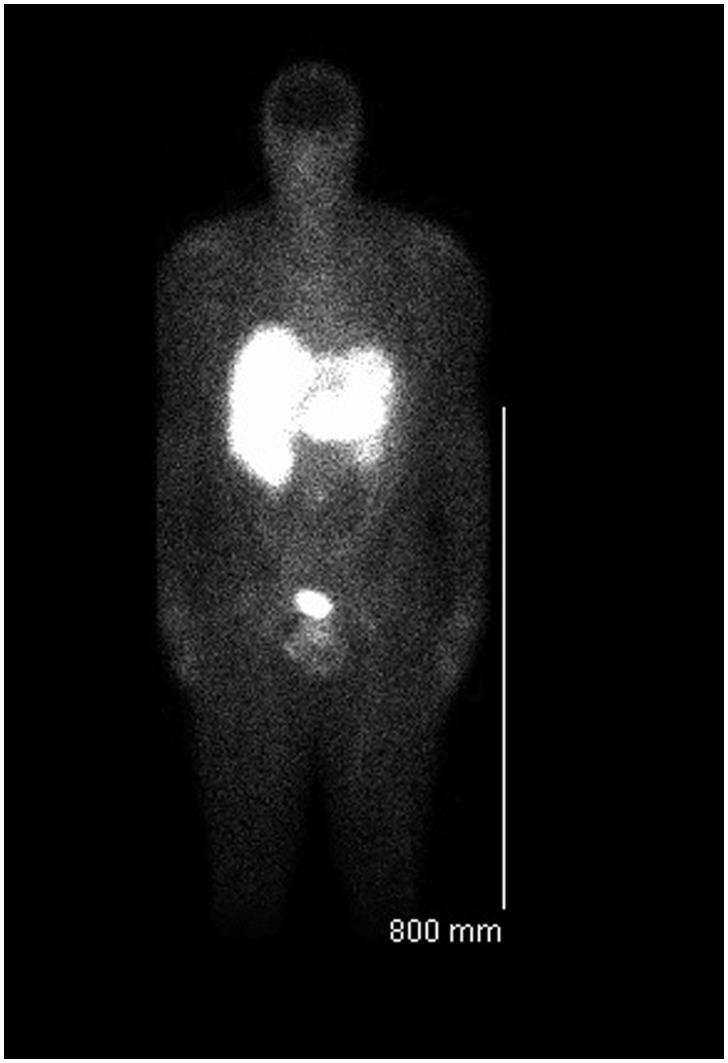
Somatostatin receptor scintigraphy image demonstrating radiotracer uptake in multiple liver metastases.

Hematoxylin and eosin (H&E) stain revealed a well-differentiated, low-grade NET (Figure 2B). Stains for chromogranin A (Figure 2C) and synaptophysin (Figure 2D) were positive, consistent with a NET. Somatostatin receptor scintigraphy (SRS) revealed increased radiotracer uptake within the liver but not in the terminal ileum, due to the small size of the tumor (Figure 3). Levels of 5-hydroxyindoleacetic acid (HIAA) were elevated, at 320 mg/day (normally 2–6 mg/day).

The patient was started on octreotide therapy and responded abruptly, with a significant decrease in bowel movements and weight gain over the following months. He was further treated with palliative chemoembolization of liver metastases in an attempt to decrease the tumor burden in the liver and relieve symptoms for a better quality of life. He remained symptom-free for 6 months, but symptoms recurred and disease progressed with metastasis to the bone. The patient underwent further palliation with radiotherapy but his long-term prognosis is poor due to the advanced stage of the cancer.

## Discussion

Neuroendocrine tumors (NETs) were first described in 1907 as arising from enterochromaffin cells in the submucosa of the intestine. The incidence of NETs has been on the rise, with recent studies reporting a rate of 4–8 per 100 000 people [1–3]. They are classified based on histological differentiation, tumor size, angioinvasion, and infiltrative growth. Approximately two-thirds of NETs arise from the GI tract [4], the majority of which remain clinically silent (i.e. ‘non-functional’ NETs), while the rest can either present with local symptoms (e.g. intestinal obstruction, bleeding or perforation) or symptoms of hormone hypersecretion (i.e. ‘functional’ NETs) [5].

The present patient was seen by several physicians over the 2-year period prior to his presentation, but hepatomegaly was overlooked on physical examination. Premature closure was probably the culprit, as the patient carried a diagnosis of irritable bowel syndrome and did not develop other symptoms that would suggest an alternate diagnosis. The first colonoscopy was also found to be incomplete, without terminal ileoscopy, and may have missed the primary tumor at an earlier stage.

The diagnosis of carcinoid syndrome is based on the clinical presentation and supported by the presence of elevated levels of serum chromogranin A and 24-hour urinary 5-HIAA, a breakdown product of serotonin. Once diagnosis is confirmed, SRS is recommended using a radioactive-labeled octreotide analogue, 111-indium pentetreotide, to localize primary tumors and identify metastasis. This imaging modality has a sensitivity of 80–90% [2, 3]. Additionally CT of the abdomen and pelvis, with intravenous contrast, is recommended for assessment of metastatic disease, mesenteric fibrosis and lymphadenopathy, as well as monitoring response to therapy [6]. The sensitivity of CT scans and MRIs for detection is around 80% [5]. Following localization, tissue diagnosis should be obtained, including performing upper endoscopy and colonoscopy with ileoscopy [5].

There are no clear guidelines for work-up of non-functional NETs, and they are usually diagnosed incidentally (e.g. during surgery for a different gastrointestinal disease, or emergency laparotomy for obstruction or perforation from local disease) or when disease metastasizes [5].

In retrospect, the location of the present patient’s tumor was consistent with the advanced stage of presentation with liver metastasis, which seems to be present in 50% of these cases. Furthermore, ileal NETs are more likely to be associated with carcinoid syndrome in the setting of liver metastases: approximately 20% of cases. The rate of 5-year survival with metastatic disease from an ileal source is poor, and can be as low as 18% [6].

The mainstay of treatment is removal of the primary tumor to achieve cure. However, patients who present with symptoms of carcinoid syndrome usually have advanced disease that is incurable. The aim at that point is palliative medical and, to a lesser extent, surgical interventions. The somatostatin analogue, octreotide, is the agent of choice for management of symptoms and is currently the standard of care for metastatic NETs. Systemic chemotherapy is another option, but has a poor response rate of 10–30%. Surgical excision of metastatic disease is an option when the lesions are smaller and limited [4]. Other cytoreductive procedures are available, such as radiofrequency ablation, laser therapy and embolization of liver metastases, all of which aim to reduce symptoms and improve quality of life [7, 8].

## Conclusion

A thorough case history and physical examination are essential in identifying patients with organic causes of chronic unresolving diarrhea. When screening for organic and functional causes of diarrhea, the possibility of a NET should be considered, as many associated symptoms of carcinoid syndrome may not be reported initially and may become apparent only on further disease progression.
